# Determinants of lifestyle and body weight status among breast cancer survivors with overweight/obesity and perspectives towards the development of weight loss interventions: a qualitative study with health professionals from Greece

**DOI:** 10.1017/jns.2023.117

**Published:** 2024-01-23

**Authors:** Georgios Saltaouras, Maria Perperidi, Dimitra Vantzou, Konstantina Vatsina, Emmanouil Saloustros, Yannis Theodorakis, Odysseas Androutsos

**Affiliations:** 1 Laboratory of Clinical Nutrition and Dietetics, Department of Nutrition and Dietetics, School of Physical Education, Sport Science and Dietetics, University of Thessaly, Trikala, Greece; 2 Department of Nutritional Sciences and Dietetics, International Hellenic University, Sindos, Greece; 3 Department of Oncology, Medical School, University Hospital of Larissa, Larissa, Greece; 4 Department of Physical Education and Sport Science, School of Physical Education, Sport Science and Dietetics, University of Thessaly, Trikala, Greece

**Keywords:** Attitudes, Breast cancer, Health professionals, Lifestyle, Obesity management

## Abstract

The current study assessed the views and attitudes of health professionals (HPs) regarding factors associated with energy balance-related health behaviours and weight management in breast cancer survivors (BCS) with overweight and obesity. Semi-structured online interviews were conducted with 21 HPs (oncologists, dietitians- nutritionists, physical education instructors, mental health professionals, and nurses) from Attica and Thessaly. Thematic analysis was used to analyse and present the data. Four main themes arose from the data: “The patients’ mental health wellbeing”, “Survivors’ interest in diet and exercise”, “Interdisciplinary collaboration in patient’s care”, and “Maintaining normality”. HPs agreed that weight loss in BCS with overweight and obesity is important, but negative mental health wellbeing is a main barrier to behaviour change. For many BCS their cancer diagnosis is a “teachable” moment for weight management, especially for women of younger age, who are more keen to discuss weight management issues. Essential characteristics that determine/facilitate behavioural change include education, commitment for regular communication, personalised intervention, and interdisciplinary collaboration. According to HPs, future weight loss interventions should take into account BCS’s mental health wellbeing and level of motivation and should provide regular support and education.

## Introduction

Female breast cancer continues to be one of the most common cancers, with an estimated 2.3 million new cases in 2020^(1)^ but also with increased 10-year survival trends.^(2)^ Increasing cancer incidence and survival act together to increase cancer prevalence, as more people are diagnosed, but at the same time more people live longer after or with cancer.^(2)^

Weight gain is a common treatment side effect among breast cancer survivors (BCS), along with chronic pain, fatigue, and reduced mobility which can further impact on weight.^(3)^ A recent systematic review and meta-analysis showed for the first time that high post-diagnosis body mass index (BMI), waist circumference, and waist-to-hip ratio were all strongly associated with increased risk of all-cause and breast-cancer-specific mortality.^(4)^ Obesity after breast cancer is also associated with increased risk for the development of chronic diseases, such as hyperlipidaemia, hypothyroidism, and other forms of cancer.^(5)^ Extensive systematic reviews have shown that weight loss interventions incorporating diet, physical activity, and psychosocial support are associated with decreased body weight and improvement in overall Quality of Life in BCS.^(6,7)^

It is, therefore, of primary importance to address obesity in women with breast cancer at all stages of the cancer trajectory. People living with cancer could be more motivated to lifestyle changes, including behaviours towards diet and physical activity.^(8)^ Research has been conducted in relation to the views and perspectives of weight management in breast cancer survivors;^(9–11)^ however, little is known regarding obesity management support in survivors of breast cancer from the health professionals’ (HP) perspective.^(12)^ HPs involved in breast cancer patients’ treatment trajectory are key contacts in communicating health messages and supporting cancer survivors in lifestyle changes.^(13)^

For the preparation of successful weight management interventions in BCS, it is essential to explore the views of both BCS and HPs involved in their care after diagnosis. The aim of this study was to assess the views and perspectives of HPs in relation to factors that affect lifestyle and weight management in BCS and the acceptability of a lifestyle intervention for weight loss.

## Methods

### Ethical approval

The study was approved by the Bioethics Committee of the Department of Nutrition-Dietetics, University of Thessaly (07/02.02.2022) and adhered to the principles of the Declaration of Helsinki and the General Data Protection Rules (GDPR).

### Study population

The study incorporated semi-structured interviews with HPs who have worked with or have been involved in the care of breast cancer patients/survivors. Purposive sampling was used to recruit a diverse sample of participants from different specialties (oncologists, nurses, dietitians-nutritionists, physical education instructors, and mental health professionals) from two counties in Greece (Attica, Thessaly).

### Procedure

An invitation letter was sent to local networks, hospitals specialised in cancer care, cancer charities, and forums. HPs who responded to the invitation were contacted for further information and an online interview was organised at the participant’s disposal. All necessary information was provided prior to the interview. An interview guide was developed by the research team (Supplementary Material 1) and was guided by a recent systematic review in behaviour change techniques in nutrition and physical activity interventions in post-treatment breast cancer survivors performed by the same research group.^(14)^ Participants provided written consent prior to the interview, which was sent to the researcher via email. It was explicitly mentioned that participation was voluntary, with the right to withdraw at any time. The interviews took place between October and December 2022 and were conducted online with the use of an electronic platform. Virtual interviews have shown to be as efficient as in-person interviews, less costly, and less time-consuming.^(15)^ The interviews were conducted by one researcher, while a second researcher was also present. Both researchers have a background in dietetics and have experience in conducting qualitative research.

To ensure rigour, the first interview was used as a pilot to test the content of the questions and ensure adequate flow of the interview. No major changes were made and the first interview was included in the analysis. At the end of each interview, a reflection diary was kept by the interviewer to ensure that their own positions and attitudes would not affect the interpretation of the content of the interviews.^(16)^ Basic demographic characteristics, such as age, gender, profession, qualifications, years of specialisation/experience, and area of employment were collected. Interviews lasted approximately 30 minutes.

The concept of “data saturation”, frequently used in qualitative research, along with the concept of capturing views from different specialties, was used to determine adequate sample size. Through reflexive summaries at the end of each interview and debriefing with all authors, the study was terminated after 21 interviews.

### Data preparation

Interviews were video recorded and transcribed verbatim. All interviews were de-identified during transcription. Electronic files and transcripts are held in password-protected computers in the Laboratory of Clinical Nutrition and Dietetics at the University of Thessaly.

### Data analysis

Thematic analysis was used to analyse the data from the interviews, according to Braun and Clarke^(17)^ as it is commonly used in qualitative research in nutrition and dietetics from non-experienced qualitative researchers.^(18)^ A combination of deductive (topic guide) and inductive approach was used in this thematic analysis. Transcripts were read by two researchers and an initial coding framework was developed after the line-by-line coding of five transcripts. Codes were then applied to the remaining interviews resulting in further changes. Codes were organised in themes and subthemes and were agreed with the whole research team.

Four main themes arose from the data: “The patient’s mental health wellbeing”, “Survivors’ interest in diet and exercise”, “Interdisciplinary collaboration in patient’s care”, and “Maintaining normality”. Themes and subthemes are presented in the results along with selected quotes that demonstrate them. The study fully complied with the Standards for Reporting Qualitative Research (SRQR; Supplementary Material 2)^(19)^

## Results

In order to reach 21 interviews and achieve data saturation, a total of 45 HPs were approached to be interviewed. In total, 45 HPs expressed interest and were contacted to participate, of which 21 agreed to be interviewed (47%). Participant demographics are presented in Table 1. Mean age was 43.4 years, with the majority having 10-20 years of experience in their field (43%). Most of the participants held a Postgraduate Degree (76%) and were employed in Attica (76%). Seven participants worked in public hospitals (two of them also freelancers), six were freelancers, four in research/clinical practice, two in charities (and freelancers), one in a private hospital, and one in a nutrition clinic. A summary of themes and subthemes is presented in Table 2.

**Table 1.** Participant characteristics (n 21)

		N (%)
Age (mean)		43.4 (7.4)^a^
Gender	Male	8 (38.1)
	Female	13 (61.9)
Profession	Oncologist	4 (19)
	Dietitian-nutritionist	5 (24)
	Nurse	3 (14)
	Physical education instructor	5 (24)
	Mental health professional	4 (19)
Health service	Public	7 (33)
	Private and/or freelance	12 (57)
	Both	2 (10)
Experience	<10 years	5 (24)
	10–20 years	9 (43)
	>20 years	7 (33)
Qualifications	Professional qualification	1 (5)
	University degree	4 (19)
	Postgraduate degree	16 (76)
Area of employment	Attica	16 (76)
	Thessaly	5 (24)

a mean (standard deviation).

**Table 2.** Themes and subthemes

Themes	The patient’s mental health wellbeing	Survivors’ interest in diet and exercise	Interdisciplinary collaboration in patient’s care	Maintaining normality
Subthemes	Negative mental health state	The diagnosis as a “teachable moment”	Together but also individually	The need to regain control
	Positive mental health state	Patient attitudes towards increased weight and obesity	The importance of education	The “power” of habit
	Food as a “reward” to overcome the disease	Decreasing adherence to a healthy diet after the end of treatments	The “therapeutic contract”/building a new behaviour	
		The role of age	The influence/power of the oncologist	

### Theme 1: the patient’s mental health wellbeing

#### Negative psychology/mental state

Most participants highlighted the negative influence of diagnosis and treatments on women’s mental health status. Survivors’ feelings of fear, shame, injustice, guilt, and stress in relation to the onset of disease were frequently mentioned by participants and were seen as major barriers to change. The negative effect of treatments on body weight added more disappointment and stress.

“*A diagnosis is a big shock, great disruption and a lot of changes a patient needs to do, so since they experience so many changes simultaneously, asking them to change their diet radically is one more change and sometimes they are in denial, “I can’t do it”, “I don’t want to do it”, “Why did this happen to me?”, “I need to change so many things in my life”. So, it is expressed as denial, or stress or even guilt*”. **(P05)**

“*When it comes to diet there is a big issue, […] because most BCS cannot follow a specific diet plan due to a negative mental health state*” **(P17)**

#### Positive psychology/mental state

According to participants, despite widespread negative feelings, some BCS express the need to focus on self-care in order to *“love themselves again”* and work on rebuilding their body. BCS aim to find ways to overcome the disease, feel strong, and carry on with their life.

“*… (BCS) are motivated to make changes in relation to mental health which includes self-care, and self-care means to take care of my soul and my body*” **(P05)**

“*…being healthy, that is to take good care of themselves again. It is not only about body image but also something more important, more internalised”* **(P12)**

#### Food as a “reward” to overcome the disease

Two participants (both dietitians and nutritionists) referred to emotional eating as a way to control negative feelings related to the disease and its treatments. Both dietitians and nutritionists focussed on the difficulties of dealing with emotional eating.

“*They tend to say “I have been through such a difficult situation and now I am told to have more restraints and boundaries. I have been told to stop eating salt and sugar during therapies and it is always difficult because I have been dealing with cancer and I believe I deserve a little sweet treat, […] I deserve this because I am so tired*” **(P11)**

### Theme 2: survivors’ interest in diet and exercise

#### The diagnosis as a “teachable moment”

According to participants, most BCS were interested and receptive to discussions around lifestyle changes, while sometimes initiated these discussions from the point of diagnosis. BCS were interested in finding out *“what they could do”* to reduce weight, to reduce the risk of recurrence, to improve Quality of Life, and to reduce the negative effects of hormone therapy such as osteoporosis and dyslipidaemias. Each group of experts had a different level of engagement to weight management conversations, with oncologists and – to a lesser degree – nurses discussing issues about diet, physical education instructors about exercise, mental health professionals about body image, and dietitians-nutritionists providing a more holistic approach.

“*From the first meeting, before we even finish talking about therapeutic targets they (BCS) ask me what they should eat and which foods have been linked with cancer and whether there is a specific diet to prevent recurrence*” **(P02)**

“*They ask about (cutting down on) sugar, they read about the role of sugar and sweets and ask if they should stop eating sugar*” **(P16)**

However, several participants mentioned that BCS often felt an obligation, rather than an opportunity, to implement lifestyle changes *(“I must change otherwise I will be in danger again”* **(P05)**). The *“obligation”* was seen as both a facilitator and a barrier towards lifestyle change.

#### Patient attitudes towards increased weight and obesity

Participants mentioned that BCS frequently experienced weight changes and understood these changes were due to the cancer itself and the associated treatments. According to them, patients frequently express the need to do something to change their body weight, particularly if changes occurred during treatments and were related to them. However, some scepticism from BCS regarding weight loss during treatments is also apparent, as it may be related to undernutrition.

“*… different therapies and especially the use of corticosteroid medication has radically changed BCS’s body image and that is a real shock*” **(P04)**

“*The main barrier […] is the contrast in these women’s beliefs. We are used to a cancer diagnosis being linked to sarcopenia, weight loss […] but in this population we have a different situation, we have women with increased weight […] so we will tell them to lose weight and they believe it will harm them, it will make them weaker*” **(P11)**

#### Decreasing adherence to a healthy diet after the end of treatments

Some participants, particularly oncologists, mentioned that despite discussions around diet and body weight, patients would not follow the advice long after the end of treatments. They believed that support from HPs does not necessarily lead to lifestyle change or body weight change.

“*I think they are concerned (about their lifestyle), especially at the beginning because hormone therapy can cause all these problems and it makes them gain weight […] but later on, when treatments go well, it (lifestyle) is less of a concern*” **(P18)**

#### The role of age

The age of the patient was viewed as an important factor towards lifestyle changes for most participants. Younger BCS were believed to be more motivated for lifestyle changes and more receptive to discussions around weight management. They would also be more interested in digital-based weight loss interventions, as they are more familiar with technology.

“*Generally, younger women are more receptive (to lifestyle changes). Older women do not accept, they are not interested and they will not ask about lifestyle. Younger women are more concerned about their weight and their Quality of Life, as well as their appearance*” **(P08)**

### Theme 3: interdisciplinary collaboration in patient’s care

#### Together but also individually

Participants highlighted the need for a holistic approach in BCS’s care and appropriate sources that can be accessed by both BCS and HPs. The need for an organised space in health clinics specially made for BCS’ care was also mentioned.

“*We have tried a lot to raise awareness to the doctors for the need (the BCS) to be referred to a mental health professional because they need it; the same goes for a dietitian […]*” **(P10)**

“*There should be a weight management clinic specific for BCS after treatments*” **(P04)**

Concurrently, participants believed that each HP has their own field of expertise i.e. a dietitian–nutritionist is the expert on nutrition and a physical education instructor on exercise. This point was particularly raised by dietitians-nutritionists, mental health professionals, and physical education instructors.

“*I am a physical education instructor and I don’t want to provide advice that are another expert’s field but I like collaborating with other experts. I may know a few things about nutrition but I am not an expert*” **(P01)**

#### The importance of education

Education of BCS on lifestyle was valued as essential for most participants, with a particular focus on the role of diet, which was highlighted not only by dietitians-nutritionists, but also other health professionals. The HP needed to be an expert on the field and would help BCS deal with the provision of simple and effective solutions in a simple language.

“*They (BCS) knew I had experience (with patients with cancer) because I had attended relevant seminars*” **(P01)**

“*There are some cases (BCS) who will have heard all these foods they should avoid because of cancer, everything they find on the internet, and we will deal with this during our discussion*” **(P04)**

#### The “therapeutic contract”/building a new behaviour

Some participants, particularly dietitians-nutritionists, stated that weight loss should be a part of cancer therapy and that it should continue after the end of cancer treatments *“After such a serious health problem, it (weight loss) should be viewed as a continuation of treatment, but it is not easy for BCS to understand that or they are not mentally prepared to accept it”* **(P04)**. The need for commitment from both parts – HPs and patients – in communication and regular meetings was expressed by several participants, who also suggested that a trusting relationship with honesty and intimacy needs to be built. Other characteristics that lead to successful collaboration included the provision of small, achievable targets and the establishment of relationship boundaries.

“*I believe that when you talk to them honestly and openly, they feel comfortable and they become more open with you as well*” **(P07)**

“*These patients had gone through a period when they needed to be consistent in their treatment appointments, […] so I use this “consistency” to set weight loss targets […] and this is motivating for them*” **(P11)**

Several participants have agreed on a personalised approach towards behaviour and lifestyle changes, due to different therapies for this cancer group and the patients’ need for flexibility. One participant (dietitian-nutritionist) disagreed, stating that BCS have similar targets to other people who need to lose weight.

“*I believe a personalised approach with every patient is useful, even in relation to mental health and diet; all these should be discussed with the patient first*” **(P08)**

“*I can’t see the difference between a BCS and a woman without a diagnosis; I deal with similar issues […] and generally with all people with overweight and obesity who do not know how to change their lifestyle*” **(P06)**

In relation to the implementation of a weight loss intervention for BCS, a combination of face-to-face and online meetings would provide the required flexibility for this population, so they achieve better adherence and change lifestyle behaviour. Some physical education instructors were sceptical for a solely online intervention for physical activity and believed BCS would benefit more from face-to-face meetings.

“*Some women may have difficulty commuting. It may be easier to have an online session and meet in person every now and then*” **(P10)**

“*I prefer a face-to-face and a one-on-one training session, so the focus of the trainer is solely on the person*” **(P13)**

#### The influence/power of the oncologist

Participants from all professions agreed that BCS have a more trusting relationship with their oncologist and would be more amenable to change if oncologists held conversations or referred them to other health professionals for weight loss. *“Some women come to me saying “My doctor said I should exercise””* **(P12)**

Some oncologists admitted that conversations on weight management is not priority and are not held routinely. Other HPs believed oncologists did not regularly discuss weight loss with BCS.

“*The doctor will say “You’ve gone through this (cancer), we need to keep an eye on it” and they will not mention anything (about diet)*” **(P09)**

“*Yes, we will discuss a few things (on nutrition), but to be honest it is not the first thing to discuss. […] If a woman is overweight, we may advice to watch their weight. But in all honesty I do not have an extensive conversation with them*” **(P16)**

### Theme 4: maintaining normality

#### The need to regain control

According to several participants, many BCS express the need to regain control of their health and ask for ways to carry on with everyday life in a way similar to prior their diagnosis. The need to maintain normality could be assisted by participation in a group-based lifestyle intervention, where survivors would be able to share their experiences and get feedback from other patients who have gone through a similar cancer trajectory.

“*When breast cancer becomes a chronic disease, you realise that a woman who is young, has a partner, family, children […] wants to have a good physical state and adopt healthy eating habits*” **(P08)**

“*Their motive is to go back to a normal lifestyle […] So it was a difficult disease, it is over now, the outcome is positive, so they are motivated because they want to leave this to the past and move forward to a better everyday life*” **(P11)**

#### The “power” of habit

Previous lifestyle habits could act as facilitators or barriers, according to participants. Pre-existing knowledge and practices of survivors in relation to diet and physical activity, as well as having excess weight prior to diagnosis would influence lifestyle changes. The experience of weight loss in the past was perceived as a barrier towards lifestyle change.

“*There are some BCS who had experience with exercise (before diagnosis), so they know that after treatments they will see improvements through exercise. So it is related to previous experience*” **(P12)**

“*They may say “I want to go out, do whatever other people do but I need to be careful” […], foods that women regularly consumed in their diet and it is very difficult for them to stop*” **(P20)**

**Fig. 1.**
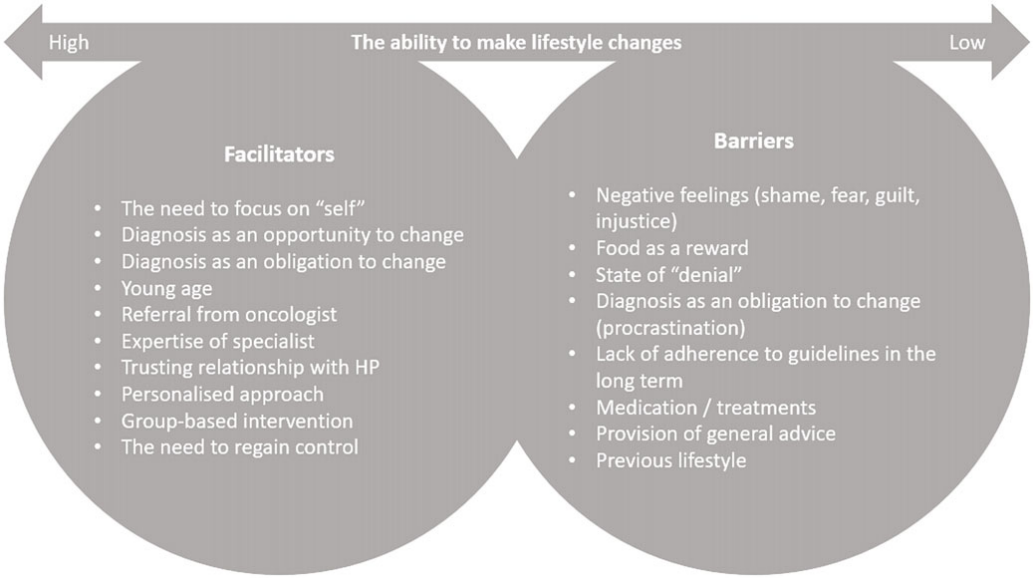
Facilitators and barriers of BCS towards making lifestyle changes, according to HPs. BCS =  breast cancer survivors; HP = health professional.

Figure 1 summarises the facilitators and barriers for BCS to make lifestyle changes after diagnosis, according to HPs.

## Discussion

This study assessed HPs’ views in relation to factors that influence lifestyle behaviours and weight management in BCS, as well as their perspectives for the development of weight-loss interventions in this population. According to the findings, HPs perceived mental health as an essential factor that contributes to health behaviours. Negative feelings and attitudes of BCS were a main barrier for lifestyle change, whereas the need of BCS to care for their health and body was a facilitator for change. BCS were generally interested in learning how to change their lifestyle and frequently asked for guidance from the point of diagnosis, as they would view the onset of the disease as an opportunity to change their lifestyle and lose weight. However, discussions would not lead BCS to lifestyle changes in the long term. The interdisciplinary collaboration between HPs was a main finding in this study, with participants stating the importance of different experts working together to improve BCS health. Influencing a new lifestyle behaviour would require trust between HPs and BCS, regular meetings, and setting of realistic goals. Finally, the need to maintain normality and carry on with life similar to prior to diagnosis shows the BCS’s need to regain control of their life and move past the disease.

In this study, HPs engaged in conversations about weight management, incorporating diet, physical activity, and/or mental health, but there was substantial variation on the level of engagement and topic of discussion between HPs working in different capacities. The role of the oncologist was further highlighted (Theme 3; subtheme 3.4) as they are viewed as the key HP to promote participation in lifestyle interventions and refer them to other weight management specialists, similar to other studies.^(20)^ However, oncologists admitted not engaging very often to lifestyle-related conversations, due to time constraints and to prioritising treatments. Barriers such as time constraints, lack of nutrition education, negative perceptions of the importance of diet and low interest in the topic of nutrition may lead to low self-confidence in providing nutritional counselling among General Practitioners.^(21)^

Specialties such as oncologists, surgeons, and nurses engage in discussions with their patients regarding weight management but may not have the required knowledge.^(22)^ In a study conducted by the American Society of Clinical Oncology, over 85% of oncologists agreed weight management support should be part of cancer treatment interventions but less than 40% had the knowledge and training to address issues related to obesity.^(23)^ A recent review on the views of medical and nursing staff in the provision of diet and exercise advice to cancer survivors highlighted the importance of such interventions and showed that they may be more confident in referring cancer survivors to appropriate health experts (i.e. dietitians-nutritionists and exercise professionals) than providing advice based on their role.^(24)^ Interdisciplinary collaboration for effective care was a main finding in this study and was also enhanced by the need to refer BCS to the right experts for effective support.

Psychosocial needs of women with breast cancer have been long investigated.^(25,26)^ According to a systematic review, BCS have higher levels of depression or depressive symptoms, anxiety or anxiety symptoms, stress, and sleep disturbances.^(27)^ Although breast cancer medical treatments have significantly advanced over the years, breast cancer care still fails to address psychosocial and Quality of Life issues and coping with late side effects and body image, which may be present long after the end of treatments.^(28)^ BCS with obesity who gain further weight during treatment experience a higher decline in health-related Quality of Life, compared to BCS with obesity who lose weight.^(29)^ It is, therefore, important to incorporate psychosocial determinants in future weight loss interventions.

The role of age was another significant finding in this study. Previous research has focussed on psychosocial needs of young BCS in which concerns about fertility, body image, employment, family relationships, and information needs have been highlighted as unmet needs.^(30)^ In our study, body image and family relationships were viewed as motives towards lifestyle change so that BCS regain normality. It is also known that younger women have a greater risk for the development of aggressive breast cancer,^(31)^ and a higher risk of 5-year local recurrence.^(32)^ Aggressiveness of disease and risk of recurrence could be factors that further motivate younger BCS to engage in weight loss interventions.

Several facilitators and barriers towards lifestyle change after breast cancer diagnosis have been identified. In the current study, the negative feelings, the effect of treatments, and the lack of long-term adherence were seen as main barriers towards lifestyle change. On the other hand, the need to get control of life and maintain normality, as well as interdisciplinary collaboration and HPs’ expertise could be useful tools in future successful interventions. Previous studies have highlighted institutional barriers (limited time, lack of referrals, lack of funding), healthcare-related barriers (lack of knowledge, low priority), and patient-related barriers (negative emotions, mental health issues, limited time and knowledge, lack of motivation and accessibility, increased costs, treatment long-term side effects, other health issues) towards the promotion of lifestyle interventions.^(9–11)^ On the other hand, facilitators of lifestyle change included structured interventions, being accountable to someone else, social support, support and education from HPs, perceived ability to self-manage, sense of fulfilment, desire to stay healthy, and improvement of mental wellbeing.^(9,11,13)^ Future lifestyle and weight loss interventions would benefit from incorporating factors that determine the patients’ ability to change.

Qualitative studies have highlighted that although cancer diagnosis is often perceived as a “teachable moment”, it does not always lead to behaviour change, as barriers can outweigh the motivation to eat healthily^(33)^ and exercise.^(34)^ Indeed, results from a recent systematic review revealed that cancer survivors made some positive dietary changes after diagnosis, such as lower total energy and lower carbohydrate consumption, but these changes were small and not clinically meaningful.^(32)^ Adherence to lifestyle recommendations (diet, exercise, body weight) is also shown to be low in women with a breast cancer diagnosis.^(35)^

It is important to note distinct differences of engagement in this qualitative study depending on participants’ profession. Dietitians, physical education instructors, and mental health professionals were very engaging in sharing their views and showed increased interest in the current study, whereas nurses were more difficult to recruit as they seemed less interested in the topic. There was mixed interest among invited and finally interviewed doctors.

## Strengths and limitations

This study has certain strengths and limitations. The main strength is that a qualitative investigation of the research questions enabled a more in-depth understanding of the experiences of HPs regarding weight management of BCS, which would not be unveiled from a quantitative research design. Rigour was ensured with the use of a pilot interview, a reflective diary by the researcher who conducted the interviews and the detailed analysis of interviews by two researchers. There are, however, several limitations. First of all, subjects were recruited through local networks, hospitals specialised in cancer care, cancer charities and forums, which may have led to sampling and selection bias. HPs who participated may have been more interested in issues around lifestyle in cancer survivorship. Interviews were conducted with the use of an online platform and it is acknowledged that this method may lead to obtaining less contextual information, compared to face-to-face interviews.^(36)^ However, there is literature to support the use of virtual interviews for flexibility and low costs without loss of significant information.^(15)^ Finally, researcher bias may have occurred, due to the researchers’ educational background (nutrition-dietetics) and the absence of a qualitative data analysis software (which would provide more reliability and control), despite close monitoring by two researchers.

## Conclusion

In conclusion, the study identified facilitators and barriers that, according to HPs, influence the ability of BCS with overweight or obesity to adopt healthy lifestyle behaviours and achieve weight loss. Consultations on body weight management and weight loss interventions should be part of the cancer survivorship care pathway and under a multidisciplinary approach. Future weight loss interventions should incorporate mental health support, education, a personalised approach and be led by HPs with extensive experience in their field. Incorporation of the views, experiences, and perspectives of HPs could lead in the implementation of successful future interventions.

## Supplementary material

For supplementary material accompanying this paper visit https://doi.org/10.1017/jns.2023.117. click here to view supplementary material
