# Supplementary material for: Determinants of lifestyle and body weight status among breast cancer survivors with overweight/obesity and perspectives towards the development of weight loss interventions: a qualitative study with health professionals from Greece
Source: J Nutr Sci. 2024 Jan 23;13:e4. doi: 10.1017/jns.2023.117 (PMC10808873; doi:10.1017/jns.2023.117)
Supplement: Supplementary file 1 [file jnssup.zip › S2048679023001179sup001.docx]

**Supplementary Material 1: Overview of interview topics.**

| Interview topics | Questions |
| --- | --- |
| Counselling / Conversations with BCS | Have you been involved in conversations about lifestyle changes?  Do you provide lifestyle counselling?  What do survivors ask (in relation to weight, diet, physical activity)? |
| Barriers in lifestyle changes | What are BCSs most concerned about after treatments in relation to lifestyle?  What do you believe are the main barriers of BCS in changing lifestyle? |
| Motives for lifestyle changes | What do you believe are the main facilitators so that BCS change their behaviour?  Have you used any practices to encourage your patients to change their lifestyle and manage their weight? |
| Suggestions for a lifestyle intervention | What do you think should be the main characteristics of a lifestyle intervention for weight loss (content, duration, frequency of meetings, use of digital means)?  Involvement of public sector (hospitals) or private sector (private clinics, freelance professionals)?  Do you use technology to get in touch with your patients?  Other suggestions |

BCS: Breast Cancer Survivors
