# Supplementary material for: Determinants of lifestyle and body weight status among breast cancer survivors with overweight/obesity and perspectives towards the development of weight loss interventions: a qualitative study with health professionals from Greece
Source: J Nutr Sci. 2024 Jan 23;13:e4. doi: 10.1017/jns.2023.117 (PMC10808873; doi:10.1017/jns.2023.117)
Supplement: Supplementary file 1 [file jnssup.zip › S2048679023001179sup002.docx]

**Supplementary Material 2: SRQR criteria for reporting qualitative research**

| **No** | **Topic** | Item |
| --- | --- | --- |
| **Title and abstract** | | |
| **S1** | **Title** | Determinants of lifestyle and body weight status among breast cancer survivors with overweight/obesity and perspectives towards the development of weight loss interventions: A **qualitative study** with health professionals (pg. 1) |
| **S2** | **Abstract** | Aim: To assess the views and attitudes of HPs regarding factors associated with energy balance related health behaviours and weight management in BCS with overweight and obesity.  Methods: Semi-structured online interviews were conducted with 21 HPs (oncologists, dietitians- nutritionists, physical education instructors, mental health professionals and nurses) from Attica and Thessaly. Thematic analysis was used to analyse and present the data.  Results: Four main themes arose from the data: “The patients’ mental health wellbeing”, “Survivors’ interest in diet and exercise”, “Interdisciplinary collaboration in patient’s care” and “Maintaining normality”. HPs agreed that weight loss in BCS with overweight and obesity is important, but negative mental health wellbeing is a main barrier to behaviour change. For many BCS their cancer diagnosis is a “teachable” moment for weight management, especially for women of younger age, who are more keen to discuss weight management issues. Essential characteristics that determine / facilitate behavioural change include education, commitment for regular communication, personalised intervention and interdisciplinary collaboration.  Conclusions: According to HPs, future weight loss interventions should take into account BCS’s mental health wellbeing and level of motivation and should provide regular support and education. (pg. 3) |
| **Introduction** | | |
| **S3** | **Problem formulation** | Detailed in introduction (pg. 4-5) |
| **S4** | **Purpose or research question** | For the preparation of successful weight management interventions in BCS, it is essential to explore the views of both BCS and HPs involved in their care after diagnosis. The aim of this study was to assess the views and perspectives of HPs in relation to factors that affect lifestyle and weight management in BCS and the acceptability of a lifestyle intervention for weight loss. (pg. 5) |
| **Methods** | | |
| **S5** | **Qualitative approach and research paradigm** | Not applicable |
| **S6** | **Researcher characteristics and reflexivity** | Use of a reflection diary to ensure that researchers’ own positions and attitudes would not affect the interpretation of the content of the interviews (pg.6) |
| **S7** | **Context** | Online interviews; Virtual interviews have shown to be as efficient as in-person interviews, less costly and less time-consuming (pg. 6) |
| **S8** | **Sampling strategy** | Purposive sampling was used; a variety of health professionals was invited to participate in order to obtain views and attitudes from all medical team members in touch with this population (pg. 5)  The concept of “data saturation”, frequently used in qualitative research, along with the concept of capturing views from different specialties, was used to determine adequate sample size; information from the reflexive diary also determined the end of recruitment. (pg.6) |
| **S9** | **Ethical issues pertaining to human subjects** | Participants provided written consent prior to participation. (pg. 5) |
| **S10** | **Data collection methods** | The study incorporated semi-structured interviews (pg. 5). The topic guide is available in the paper (Supplementary material) |
| **S11** | **Data collection instruments and technologies** | An interview topic guide was developed. The first interview was used as a pilot to test the content of the questions and ensure adequate flow of the interview. No major changes were made and the first interview was included in the analysis. (pg. 6) |
| **S12** | **Units of study** | 47% participation rate (results section); 21 participants were interviewed (pg. 7) |
| **S13** | **Data processing** | Interviews were video recorded and transcribed verbatim. All interviews were de-identified during transcription. Electronic files and transcripts are held in password-protected computers in the Laboratory of Nutrition and Clinical Dietetics at the University of Thessaly. (pg. 6) |
| **S14** | **Data analysis** | Thematic analysis guided by Braun & Clarke (pg. 6) |
| **S15** | **Techniques to enhance trustworthiness** | Transcripts were read by two researchers and an initial coding framework was developed after the line-by-line coding of five transcripts. Codes were then applied to the remaining interviews resulting in further changes. Codes were organised in themes and subthemes and were agreed with the whole research team. (pg. 7) |
| **Results/findings** | | |
| **S16** | **Synthesis and interpretation** | 4 themes: “The patient’s mental health wellbeing”, “Survivors’ interest in diet and exercise”, “Interdisciplinary collaboration in patient’s care” and “Maintaining normality”; 13 subthemes (pg. 6 / methods; pg. 8-18) |
| **S17** | **Links to empirical data** | 1-2 Quotes per subtheme (pg. 8-18) |
| **Discussion** | | |
| **S18** | **Integration with prior work, implications, transferability, and contribution(s) to the field** | Pg. 18-22 |
| **S19** | **Limitations** | First of all, subjects were recruited through local networks, hospitals specialised in cancer care, cancer charities and forums, which may have led to sampling and selection bias. HPs who participated may have been more interested in issues around lifestyle in cancer survivorship. Interviews were conducted with the use of an online platform and it is acknowledged that this method may lead to obtaining less contextual information, compared to face-to-face interviews. However, there is literature to support the use of virtual interviews for flexibility and low costs without loss of significant information. Finally, researcher bias may have occurred, due to the researchers’ educational background (nutrition-dietetics) and the absence of a qualitative data analysis software, despite close monitoring by two researchers. (pg. 22) |
| **Other** | | |
| **S20** | **Conflicts of interest** | None (pg 23) |
| **S21** | **Funding** | None (pg 23) |
